# Association of pituitary neuroendocrine tumors and neurofibromatosis type 1: assessing causation versus coincidence. Case report

**DOI:** 10.3389/fendo.2025.1483305

**Published:** 2025-02-04

**Authors:** Mercedes Aguilar-Soto, Julia M. Zuarth-Vázquez, Laura Leyva-Figueroa, Karla Zarco-Ávila, Armando Gamboa-Domínguez, Aldo Eguiluz-Melendez, Laura C. Hernández-Ramírez

**Affiliations:** ^1^ Department of Endocrinology, Instituto Nacional de Ciencias Médicas y Nutrición Salvador Zubirán, Mexico City, Mexico; ^2^ Department of Pathology, Instituto Nacional de Ciencias Médicas y Nutrición Salvador Zubirán, Mexico City, Mexico; ^3^ Department of Neurosurgery, Instituto Nacional de Ciencias Médicas y Nutrición Salvador Zubirán, Mexico City, Mexico; ^4^ Red de Apoyo a la Investigación, Coordinación de la Investigación Científica, Universidad Nacional Autónoma de México e Instituto Nacional de Ciencias Médicas y Nutrición Salvador Zubirán, Mexico City, Mexico

**Keywords:** endocrine neoplasia, genetic diagnosis, neurofibromatosis type 1, NF1, pituitary neuroendocrine tumors, tumor suppressor

## Abstract

**Introduction:**

Patients with neurofibromatosis type 1 (NF1) are at risk for developing various neoplasms. Since the early twentieth century, multiple cases of pituitary neuroendocrine tumors (PitNETs) occurring in this context have been published. Yet, the role of *NF1* (17q11.2) loss-of-function (LOF) variants in pituitary tumorigenesis remains unclear.

**Aim:**

We report the clinical and molecular characterization of a case of PitNET diagnosed in a patient with NF1. We also review the available data for and against a causal association between *NF1* defects and pituitary tumors.

**Methods:**

Our patient was recruited via an ongoing prospective study of individuals with neuroendocrine neoplasms. Genetic testing was carried out by means of targeted next generation sequencing (NGS) and Sanger sequencing in blood and tumor DNA, respectively. *NF1* expression was analyzed via quantitative polymerase chain reaction (qPCR) in blood and tumor cDNA. Similar cases were searched in the literature.

**Results:**

A 54-year-old-man was incidentally diagnosed with a clinically non-functioning PitNET via brain imaging. He had a personal and family history of NF1 and carried the germline pathogenic variant *NF1* (NM_001042492.3): c.147C>A, p.Y49*. Via transsphenoidal surgery, a 16 mm lesion was resected, showing strong granular cytoplasmic immunoreactivity with patchy distribution for NF1 and preserved heterozygosity for the *NF1* defect. Additional NGS ruled out germline defects in PitNET-associated genes. By qPCR, *NF1* was significantly overexpressed in the tumor when compared with another NF-PitNET, but not when compared with a corticotropinoma. We reviewed twenty-three case reports of PitNETs occurring in patients with either clinical NF1 without genetic study, individuals with *NF1* germline variants with or without clinical NF1 or associated with somatic *NF1* defects. Predominance of GH-secreting and large PitNETs, with young-onset in around half of the cases, were noticed. Two individuals developed multiple endocrine neoplasia-like phenotypes but tested negative for other relevant genetic defects.

**Conclusions:**

Although the association of NF1 and PitNETs could be coincidental, the clinical characteristics of the reviewed cases differ from those of typical incidentalomas. *NF1* could drive pituitary tumorigenesis via haploinsufficiency, but this hypothesis requires further research. Additional clinical and molecular data from large cohorts of affected individuals should help clarify this question.

## Introduction

Neurofibromatosis type (NF1) or Von Recklinghausen’s disease (MIM 162200) is an autosomal dominant syndrome predisposing patients to the development of benign and malignant tumors (1). Although the disease mainly affects the nervous system, several other organs such as the skin, cardiovascular, skeletal, and endocrine systems can be affected. It is one of the most common inherited disorders, with an estimated prevalence ranging from 1/3000-1/4000, which might be underestimated in countries without genetic testing protocols (2–4). NF1 patients have a reduction of 10-15 years in life expectancy compared with the general population (4). Germline loss-of-function (LOF) variants of *NF1* (17q11.2) underlie this phenotype in most cases, occurring *de novo* in 42% (1). Cutaneous manifestations such as *café-au-lait* spots and neurofibromas, including plexiform neurofibromas, are hallmarks of NF1 (5). Optic gliomas, Lisch nodules, choroidal abnormalities, and skeletal dysplasia are also common features (6, 7). Fifteen to twenty percent of patients develop glial low-grade tumors, predominantly in the optic pathways, the brainstem, and the cerebellum. In adults, the risk of high-grade gliomas, including glioblastomas, is increased by 10-50-fold compared with the general population (7).

Multiple endocrine manifestations have been associated with NF1 (8). Pheochromocytomas and paragangliomas (PPGLs) occur in up to 6.6% of NF1 patients, while germline and somatic *NF1* variants are detected in 3% and one-fourth of sporadic cases of PPGLs, respectively (9–13). Gastrointestinal neuroendocrine neoplasms of the periampullary region, usually somatostatinomas, are diagnosed in 1% of NF1 patients (14). Gastrointestinal stromal cell tumors can rarely occur. The association of *NF1* LOF with other endocrine neoplasms is uncertain.

NF1 has been largely associated with central precocious puberty, with a reported frequency of 2.4-5.6% (15–18). Most, but not all of these cases are associated with optic pathway tumors. Growth hormone (GH) excess occurs in 6-11% of children with NF1 and optic pathway tumors, either with or without precocious puberty (19–21). The mechanism causing GH excess is unclear, but the most accepted hypothesis involves loss of somatostatinergic inhibition from the optic pathway tumors (22). A recent study, however, described a heterogeneous spectrum of structural defects among individuals with NF1 and GH excess (23). Out of ten patients reported, six had optic pathway tumors, one of which had also a pituitary neuroendocrine tumor (PitNET), which was negative for GH immunostaining. Another patient was diagnosed with possible pituitary hyperplasia and therefore did not undergo surgery. PitNETs were also documented in two patients without optic pathway tumors. These findings suggest that PitNETs or hyperplasia might have a role in GH excess in patients with NF1.

Indeed, multiple cases of PitNETs have been reported in patients with NF1 since the early 1900s. Initially thought as a rare and possibly coincidental association, the growing number of publications in the recent years, as well as the finding of somatic *NF1* variants in sporadic PitNETs, suggest that a linking mechanism might exist between both diseases (24). Here, we present a patient with NF1 who developed a clinically non-functioning PitNET (NF-PitNET). Detailed molecular studies in this case and a thorough compilation of similar cases reported in the literature are presented.

## Case report

**Figure 1:**
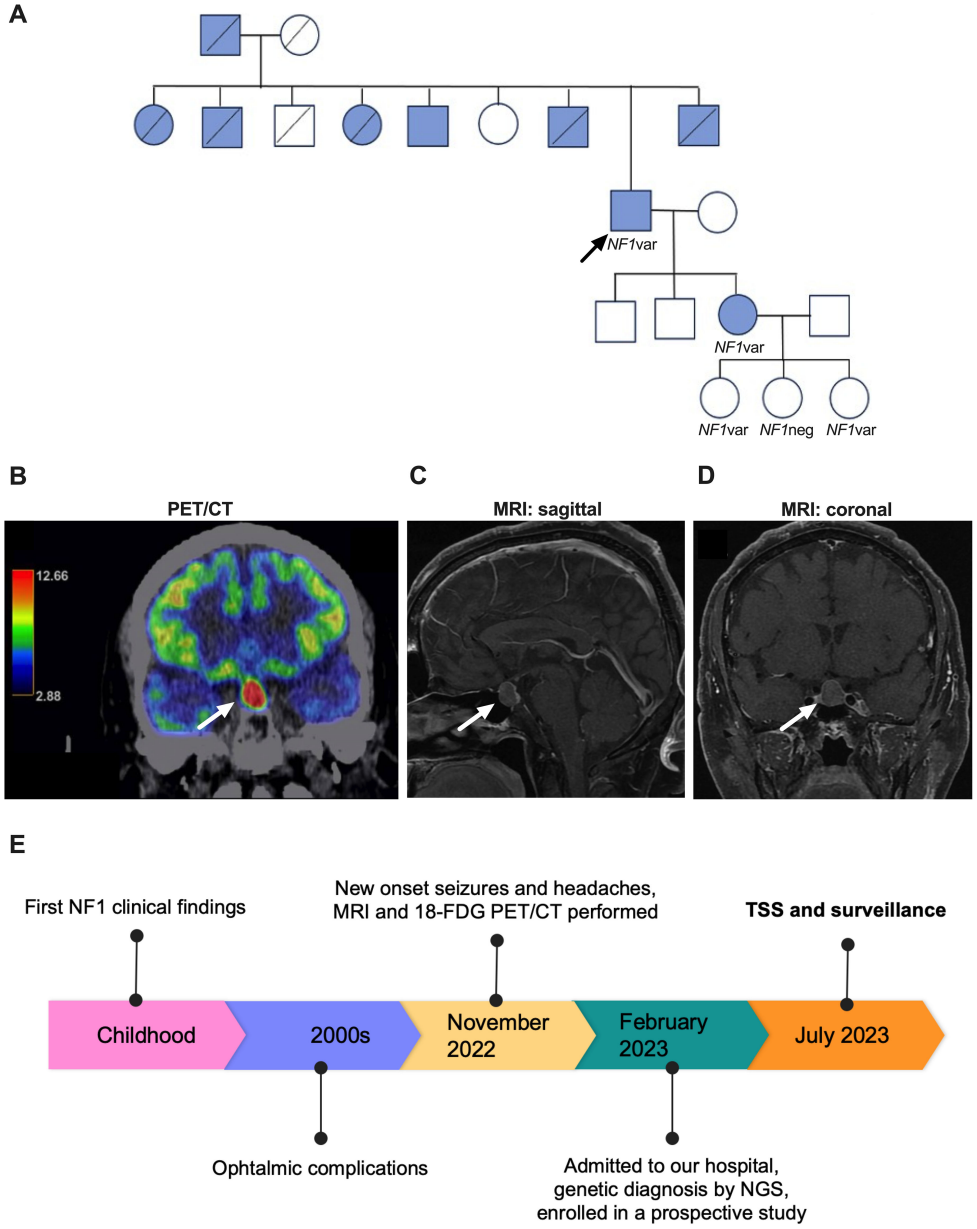
**(A)** Family tree with blue circles representing individuals affected with NF1. *NF1*var: *NF1* variant carrier. *NF1*neg: *NF1* variant screening negative. Arrow: proband. Crossed figures: deceased. **(B)** 18F-FDG PET/CT showing a pituitary lesion of with homogenous uptake of the contrast with a maximum standardized uptake value of 20.8. **(C, D)** sagittal and coronal gadolinium-enhanced MRI (T1), showing a 16x15x11 mm pituitary lesion on the left parasagittal aspect, contacting the wall of the cavernous sinus, with homogeneous enhancement and displacement of the pituitary stalk to the left and of the optic chiasm anteriorly. Arrowheads point to the lesion. **(E)** Timeline of patient evolution. TSS, transsphenoidal surgery; NGS, next generation sequencing.

A fifty-four-year-old man from San Juan del Río, Querétaro, a small city in central Mexico, was admitted to our Institute in 2023. His paternal grandmother died of an unspecified cancer and his mother died at age 48 years due to an unknown cause. He mentioned the presence of *café-au-lait* spots in his father (who died at age 85 years), eight of his ten siblings (three died before age 65 years), and his 29-year-old daughter (alive). One brother had primary biliary cirrhosis, and a nephew died at age 28 years from complications of epilepsy (**Figure 1A**). Consanguinity was mentioned. His past medical history was remarkable for the presence of NF1 stigmata (*café-au-lait* spots and cutaneous neurofibromas) since childhood, right corneal transplantation, and amaurosis of the same eye due to retinal detachment twenty years prior, well-controlled hypertension, prediabetes, dyslipidemia, and pernicious anemia.

Four months prior to admission, the patient developed short-term memory impairment, holocranial headaches, and new-onset seizures. These symptoms prompted the indication for brain magnetic resonance imaging (MRI) and 18F-fluorodeoxyglucose positron emission tomography/computed tomography (18F-FDG PET/CT), both revealing an incidental pituitary lesion; a pituitary MRI confirmed the diagnosis (**Figures 1B–D**). Laboratory tests revealed hyposomatotropinemia, but other pituitary hormones were normal (**Table 1**). On physical examination, hemianopsia of the left eye (which was the one with preserved vision) was evident by campimetry. He had multiple neurofibromas on the neck (0.5 cm), thorax, and limbs; the largest one (3x5 cm) was found in his lumbar region. The presence of multiple *café-au-lait* spots with positive Crowe sign was noted. He had a BMI of 30.2 kg/m² and his vital signs were normal.

**Table 1:** Laboratory results.

Analytes (units)	Presurgical (February 17, 2023)	Postsurgical (September 18, 2023)	Normal range
TSH (mIU/ml)	1.79	2.63	0.3-5
FT4 (ng/dl)	0.9	0.84	0.63-1.34
LH (mIU/ml)	3.55	3.34	1.24-8.62
FSH (mIU/ml)	9.33	8.84	1.27-19.26
Testosterone (ng/ml)	3.81	3.97	1.50 - 6.84
IGF-1 (ng/ml)	39.32	38.01	64-214
Prolactin (ng/ml)	12.19	7.55	3.9-29.5
Cortisol (µg/dl)	13.85	10.34	6.7-22.6
ACTH (pg/ml)	9	24	10-50
Chloride (mmol/l)	106.7	110.4	98-107
Calcium (mg/dl)	9.16	9.80	8.6-10.3
Phosphorus (mg/dl)	2.89	2.88	2.5-5
Magnesium (mg/dl)	2.04	1.89	1.9-2.7
Glucose (mg/dl)	94	101	70-99
BUN (mg/dl)	17.3	19	7-25
Urea (mg/dl)	37.02	40.66	15-53.5
Creatinine (mg/dl)	0.77	0.82	0.7 - 1.3
Cholesterol (mg/dl)	114	109	<200
LDL (mg/dl)	60	62	<130
HDL (mg/dl)	19	17	40-60
Triglycerides (mg/dl)	339	320	<150

**Figure 2:**
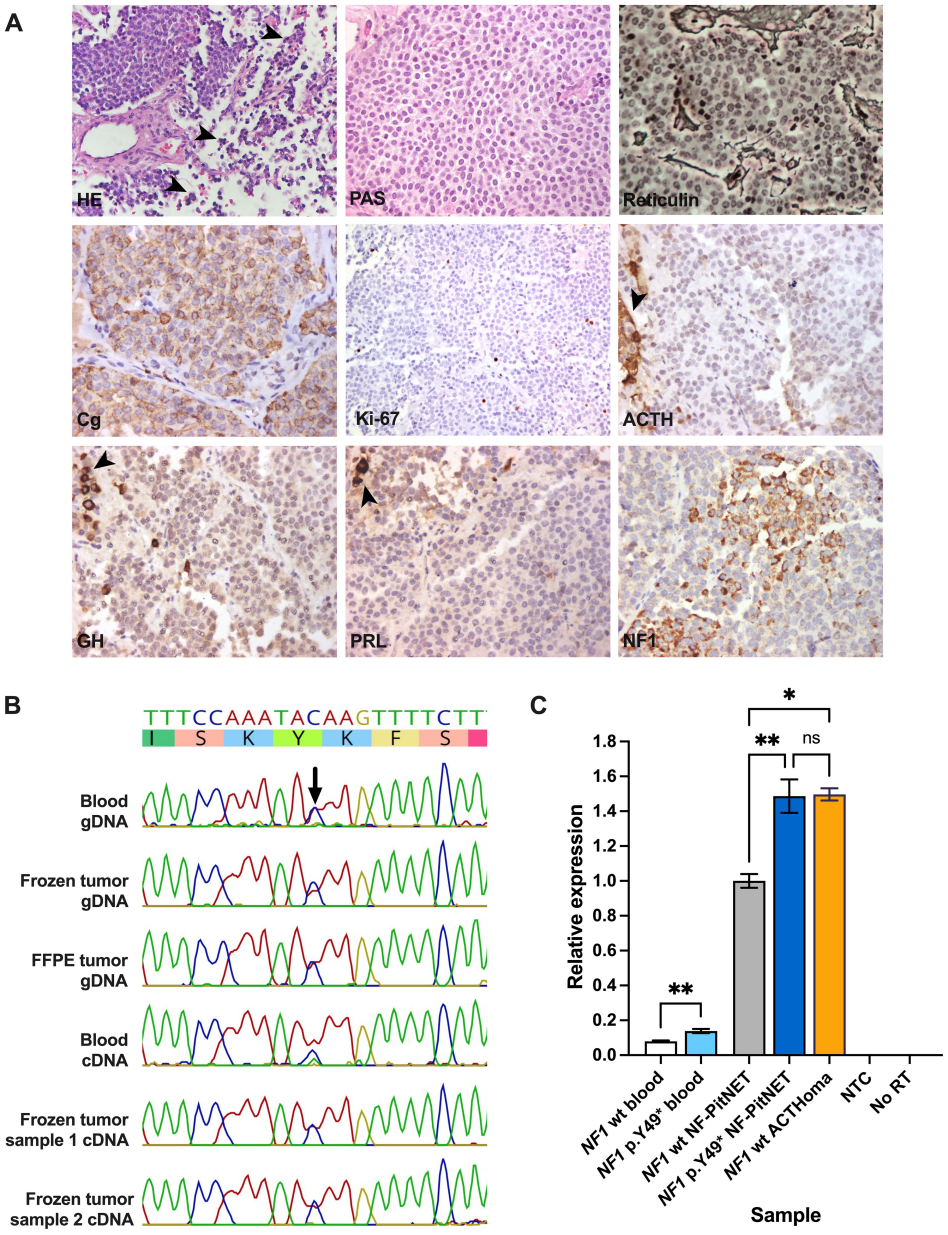
**(A)** Histopathological images of the resected PitNET. Groups of normal anterior pituitary cells surrounding the tumor (arrowheads) are shown in images for HE and negative ACTH, GH, and PRL immunostaining. Routine preparations were retrieved from the pathology archive, except for NF1 immunostaining. Magnification: 40x. **(B)** Sanger sequencing results of multiple blood and PitNET genomic and coding DNA samples from the patient. **(C)** Quantitative polymerase chain reaction depicting different blood and PitNET samples. ACTH, adrenocorticotropic hormone; ACTHoma, corticotropinoma; cDNA, coding DNA; Cg, chromogranin; FFPE, formalin-fixed paraffin-embedded; gDNA, genomic DNA; GH, growth hormone; HE, hematoxylin-eosin; NTC, no template control; *NF1* wt, *NF1* wild type; No RT, no reverse transcriptase control; PRL, prolactin. PAS, periodic acid-Schiff staining. ns, not significant; *, P≤0.05; **, P≤0.01.

Because of important visual impairment, an endonasal endoscopic transsphenoidal surgery was performed without complications (**Figure 1E**). Surgical pathology reported a PitNET with 0 mitoses per 2 mm^2^, weak, hypogranular periodic acid-Schiff staining and reticulin demonstrating focal loss of the usual acinar pattern. Immunostaining was positive for chromogranin and negative for ACTH, GH, and prolactin, and Ki-67 was <1%. Immunostaining for other pituitary hormones and transcription factors was unavailable (**Figure 2A**). Significant visual improvement was documented after surgery, although with a tumor remnant; hormonal levels remained unchanged. A diagnosis of idiopathic epilepsy was established, and the patient is currently under treatment with levetiracetam and valproic acid.

A blood sample of the patient was first studied in a routine diagnostic laboratory via a next-generation sequencing (NGS) panel (Invitae Common Hereditary Cancers Panel). This test detected a pathogenic germline variant in *NF1* (NM_001042492.3): c.147C>A, p.Y49* (dbSNP: rs1597626026, ClinVar accession: VCV002114679.2). This change, which is absent from Genome Aggregation Database v.4.1.0 (gnomAD) and the Mexico City Prospective Study (MCPS) databases, creates a premature stop codon in the exon 2, predictably leading to LOF (22, 24–26). The same variant has been reported in the literature as a germline change in two individuals with clinical NF1 (27, 28) and at the somatic level in one case of ovarian cancer (29). This defect was also reported as a somatic change in a patient with synchronous endometrial and ovarian cancer, although the germline sequence was not reported (30, 31). Two other germline variants affecting the same position and causing the same effect on the protein (c.147C>G and c.147del) have also been found in the context of NF1 (2, 5, 32, 33). The patient received genetic counseling and cascade screening for his relatives. One daughter, aged 31 years, underwent clinical evaluation and genetic confirmation. Two of the patient’s three granddaughters (aged eight and three years) have also been genetically confirmed, while the other two sons have not yet been tested.

## Experimental evaluation

### Materials and methods

Since PitNETs are not among the neoplasms typically associated with NF1, this patient was enrolled in an ongoing prospective study aimed to characterize the genetic bases of neuroendocrine neoplasms in a cohort of Mexican patients. The protocol has been approved by the internal review boards of the participating institutions (ClinicalTrials.gov NCT06523582). Under informed consent, blood samples for DNA and RNA, as well as fresh-frozen and formalin-fixed and paraffin-embedded PitNET samples were obtained. Samples from additional participants from the same study were used for comparison. Sanger sequencing was performed at the National Autonomous University of Mexico’s Research Support Network Molecular Biology unit. The rest of the experimental procedures were carried out in our lab.

DNA was extracted from blood using the DNA Isolation Kit for Mammalian Blood (Roche 11667327001) plus RNase (Roche 11119915001) treatment. For DNA extraction from tumors, samples were first mechanically disrupted with magnetic beads (fresh-frozen samples) or deparaffinized with HistoChoice (Merck H2779) and then processed with the Maga Zorb DNA Mini-Prep kit (Promega MB1004). Samples for RNA extraction were lysed with magnetic beads or red cell lysis buffer (0.1mM EDTA, 10 mM potassium bicarbonate, and 168 mM ammonium chloride), as appropriate, and further processed with the RNeasy Plus Mini Kit (QIAGEN 74134) plus DNase (QIAGEN 79254) treatment. Reverse transcription of RNA samples was done with the SuperScript III First-Strand Synthesis SuperMix for qRT-PCR kit (Invitrogen 11752050).

For NGS we used a custom-designed panel (details available on request). Library preparation was done with mechanical fragmentation and the Twist Biosciences target enrichment protocol. Sequencing was carried out in a MiSeq (Illumina) instrument, obtaining paired-end 150 bp reads with an average depth of 257x for the regions of interest. Sequencing quality was verified using FastQC, sequences were aligned to the GRCh38/hg38 human genome using Burrows-Wheeler aligner-maximum exact matches, PCR duplicates were marked with MarkDuplicates and FreeBayes was used for variant calling (34–37). Copy number variants were searched for with ExomeDepth (38).

Using the Franklin (Genoox) online platform, medium or high-quality nonsynonymous variants in exons and exon-intron junctions were identified (39). Variants with frequency <0.1% in both gnomAD and MCPS were then selected (25, 26). Variants of interest were searched for in ClinVar and their reported classification (according to the criteria of the American College of Medical Genetics and Genomics and Association for Molecular Pathology) was noted (40, 41). For variants with conflicting classification or not listed in ClinVar, functional effects were predicted using the tools linked to the Varsome online platform and data reported in the literature, when available (42). Aside from the previously known *NF1* defect, no variants of interest were identified.

To analyze NF1 expression by immunostaining, a mouse polyclonal anti-NF1 antibody (Novus Biologicals NBP2-37914, 1:200) was used, following a previously reported protocol (23). For Sanger sequencing in blood and tumor DNA and cDNA samples, a 105 bp region in exon 2 was amplified using GoTaq Green Master Mix (Promega M7122) and the primers 5’-ACAGGACAGCAGAACACACA-3’ and 5’-AGTGAGGCCGCTTATAACCA-3’. Amplicons were column-purified (Wizard SV Gel and PCR Clean-Up System, Promega A9281) and subjected to unidirectional sequencing (BigDye Terminator 3.1 Cycle Sequencing Kit, Applied Biosystems 4337456) in a 3500xL Genetic Analyzer (Applied Biosystems). Sequences were analyzed using the Geneious Prime v.2024.0.5 (Biomatters, Ltd.) software. For quantitative polymerase chain reaction (qPCR) in blood and tumor samples, 10 µl reactions were prepared using 5 ng cDNA, 1X TaqMan Fast Advanced Master Mix (Applied Biosystems 4444557), and 1X TaqMan Hs01035108_m1 FAM (Applied Biosystems 4453320) and *ACTB* VIC (Applied Biosystems 4325788) assays. Reactions were prepared in triplicates for all samples and analyzed in a StepOnePlus Real-Time PCR Systems (Applied Biosystems). Relative expression (comparative Ct method) was analyzed with unpaired t test or one-way ANOVA, as appropriate.

### Results

First, we ruled out germline variants in other genes with confirmed or suggested association with PitNETs (*AIP, ATRX, BRAF, CABLES1, CDKN1B, DICER1, CDKN1A, GNAS, GPR101, MAX, MEN1, MLH1, MSH2, MSH6, PRKAR1A, PIK3CA, PMS2, PTEN, RASD1, RET, SDHA, SDHAF2, SDHB, SDHC, SDHD, TMEM127, TP53, TSC1, TSC2, USP8, USP48, VHL*). Using Sanger sequencing, the *NF1* variant was observed in heterozygosis in genomic and coding DNA from blood and fresh-frozen and formalin-fixed paraffin-embedded PitNET tissue (**Figure 2B**). By immunostaining, the tumor displayed areas of moderate or strong granular cytoplasmic immunoreactivity for NF1 with a patchy distribution, similar to what was observed in two PitNETs from a previous publication (23) (**Figure 2A**).

Using qPCR, we observed significantly increased relative *NF1* expression in the patient’s blood (one sample) and fresh frozen PitNET (average of two samples) compared with samples from *NF1* wild type controls (*P*=0.0099 and 0.0040, respectively). *NF1* expression, however, was not increased in the patient’s tumor when compared with a sporadic corticotropinoma (*P*>0.9999) that was negative for *USP8*, *USP48*, and *BRAF* hotspot variants (**Figure 2C**).

### Discussion

We report the case of a 54-year-old man with clinical manifestations and genetic confirmation of NF1, who was diagnosed with a clinically NF-PitNET. The tumor had preserved heterozygosity at the variant locus, and the abnormal mRNA did not undergo nonsense-mediated RNA decay. By qPCR, significantly elevated *NF1* expression levels were observed in both the patient’s blood and PitNET, compared with *NF1* wild type controls, but not when compared with a sporadic corticotropinoma. The increased *NF1* expression in the tumor may be attributed to compensation for haploinsufficiency, but could also indicate a generic tumor response, since *NF1* mRNA is indeed expressed in all PitNET subtypes (43).

Aside from this case, multiple instances of PitNETs have been documented in patients with NF1. Our literature search identified 24 cases (including this one) of PitNETs in individuals with established clinical and/or genetic diagnoses of NF1 or with somatic *NF1* variants (**Table 2**). Only reports with a PitNET demonstrated by imaging studies and/or histopathological analysis were included. The earliest documented cases date back to 1912, and among those with comprehensive data available, there are records of eight females aged 7-70 years at diagnosis, and fourteen males aged 5-65 years. The reports include thirteen patients with clinical features of NF1 without genetic confirmation, eight with germline *NF1* variants (one without NF1 manifestations) and three PitNETs harboring somatic *NF1* variants. Only one patient with GH excess was reported to have coexistent optic pathway gliomas (Case 17).

**Table 2:** Previously reported cases of the association of PitNETs with clinical NF1 and/or *NF1* variants.

Case no., ref. (year)	Age*, sex	Clinical and biochemical data	Image studies	PitNET description	Genetic data**
Clinical NF1 with no genetic confirmation					
1 (55) (1912)	33 y, male	NF1 (at age 17 67 plexiform neurofibromas), glucosuria, headaches and acromegalic features (clinical diagnosis). FH unremarkable	None	Somatotropinoma?***	None
2 (56) (1912)	24 y, ns	NF1 (numerous cutaneous tumors) and acromegaly (clinical diagnosis). FH not mentioned	X-ray: enlarged sella	Somatotropinoma?***	None
3 (57) (1920)	ns, ns	NF1 and acromegaly (clinical diagnosis). FH not mentioned	None	Somatotropinoma?***	None
4 (58) (1922)	15 y, male	NF1 (soft subcutaneous nodules), obesity, glucose intolerance, precocious puberty, clinical suspicion of acromegaly and diabetes insipidus. FH unremarkable	X-ray: small shadow between the anterior and posterior clinoid processes	Somatotropinoma?***	None
5 (59) (1925)	ns, male	NF1 (multiple cutaneous tumors), acromegaly and diabetes insipidus (clinical diagnosis). FH not mentioned	X-ray: enlarged sella	Somatotropinoma?***	None
6 (60) (1970)	57, female	NF1 (neurofibromas and cerebellar astrocytoma), PitNET with hypopituitarism. FH not mentioned	X-ray: enlarged sella with destroyed dorsum and posterior clinoid processes	Chromophobe pituitary tumor	None
7 (61) (1979)	65, male	NF1 (cutaneous manifestations) and acromegaly concurrently diagnosed. Declined surgery. FH not mentioned	X-ray: enlarged sella. Carotid angiogram: elevation of the first segment of the anterior cerebellar artery, indicative of an intrasellar mass with suprasellar extension	Somatotropinoma?***	None
8 (62) (1979)	35, female	Overlapping features of NF1 (cutaneous neurofibromas and *café-au-lait* spots since age 7 y) and NF2 (meningiomas, vestibular and spinal schwannomas), and a mediastinal ganglioneuroma (associated with both NF1 and NF2). Died from pneumonia (age 35 y); PitNET found in autopsy. FH unremarkable	CT: parasagittal and subtemporal frontal, and right pterional lesions (meningiomas). Carotid angiography: parasagittal frontoparietal expansive process with bilateral extrinsic extension involving the superior longitudinal sinus	5 mm acidophilic pituitary adenoma	None
9 (63) (1980)	32 y, female	NF1 (cutaneous manifestations) and amenorrhea and galactorrhea with hyperprolactinemia and concurrently diagnosed. Treated with BEC. FH of NF1	Initial X-ray: normal. Sellar polytomography (5 y after diagnosis): bony erosion of the floor and the anterior wall of the dorsum sella by a density located on the right anteroinferior sella, consistent with an intrasellar mass. CT: no suprasellar extension	Prolactinoma?***	None
10 (64) (1990)	52 y, male	NF1 (multiple extramedullary spinal neurinomas since age 43 y). Incidental finding of PitNET with hyperprolactinemia. Underwent TSS (partial resection). FH of NF1	MRI: intra and suprasellar tumor, isointense on T1 and hyperintense on T2-weighted images	Chromophobe adenoma	None
11 (65) (2002)	49 y, male	NF1 (cutaneous manifestations since age 20 y). Presented with bitemporal hemianopsia. Underwent craniotomy. FH of NF1	CT and MRI: intrasellar lesion with a cystic portion in the suprasellar region	Clinically silent corticotropinoma	None
12 (66) (2021)	17 y, female	NF1 (bilateral orbital neurofibromas) and hyperprolactinemia. No treatment; lost to follow-up during the COVID-19 pandemic. FH not mentioned	Brain and orbits MRI: 12x12 mm PitNET	Prolactinoma?***	None
13 (67) (2021)	70 y, female	NF1 (cutaneous manifestations at age 28 y) and PHEO (48 y). Presented with concurrent PHPT, MNG, and acromegaly (IGF-1 1.7xULN, unsuppressed GH in OGTT). Underwent TSS. FH not mentioned	Pituitary MRI: 10.9x6.7 mm anterior pituitary tumor adjacent to the right internal carotid artery	Somatotropinoma: eosinophilic adenoma, diffuse positivity for GH, CAM5.2 with densely granulated pattern, Ki-67 + in scarce cells, MIB-index 0.2%	Sanger: *MEN1, RET* (exons 5, 8, 10, 11, 13, 14, 15, and 16)*, VHL, CDKN1B* (exons 1 and 2), and *CDKN2C* with no variants. *NF1* not tested
Germline *NF1* variants					
14 (68) (2013)	42 y, female	NF1 (cutaneous manifestations). History of MNG. Galactorrhea with hyperprolactinemia, acromegaly (IGF-1: 1.4xULN and OGTT with paradoxical GH increase). Underwent TSS. One daughter with NF1 (variant carrier)	Pituitary MRI: 7 mm lesion on the right side of the sella, with slight bulging of sellar diaphragm	Somatotropinoma: GH + in 100% of cells, PRL + in 5% and TSH + in 1%; FSH, LH, and ACTH -	*NF1:* c.586+5G>A, p.? (ClinVar: VCV000404473.5, pathogenic)
15 (69) (2017)	12 y, male	NF1 (cutaneous manifestations since age 7 y). Presented with headaches, blurred vision, and mild learning difficulties. FH not mentioned	MRI: 33x24x26 mm, well-circumscribed and uniformly enhancing lobulated pituitary mass eroding the pituitary floor, with superior displacement of the optic chiasm	Clinically silent corticotropinoma: prominent chromophobe hemosiderin staining and stromal fibrosis consistent with previous hemorrhage. Large subset of ACTH + cells; TSH, LH, FSH, GH and PRL -	Positive genetic test, variant not reported
16 (70) (2019)	63 y, female	Acromegaly (clinically since age 48 y, IGF-1 +5.7 SD, unsuppressed GH in OGTT). NF1 (diagnosis at age 58 y). History of bladder PGL and retroperitoneal fibrosis (54 y), abdominal aneurysm (61 y), FTC, and PHPT (one adenoma), Treated with lanreotide and then TSS. FH of NF1	Pituitary MRI: 5 mm tumor on the right side of the pituitary gland	Somatotropinoma: GH +, CAM5.2 with mixed sparsely and densely granulated pattern, heterogeneous E-cadherin staining, high SSTR2A and SSTR5 expression, Ki-67<1%	Sanger: *NF1:* c.4600C>T, p.R1534* (ClinVar VCV000220152.71, pathogenic/LP); no LOH in somatotropinoma, parathyroid adenoma, and FTC. Heterozygous somatic *GNAS* (NM_000516.7): c.601C>T, p.R201C (pathogenic) in somatotropinoma. Negative for germline *MEN1* variants, somatic *CDKN1B* variants in parathyroid adenoma, and somatic *PAX8/PPARG* rearrangements in FTC
17 (23) (2022)	5 y, male	NF1 (diagnosed at age 2 y) and gigantism since age 3 y. Underwent TSS. FH not mentioned	Pituitary MRI: optic and hypothalamic lesions, likely representing gliomas and a 4 mm right pituitary tumor	Non-functioning pituitary tumor: GH-, NF1 +	*NF1* MLPA: *NF1:* c.(576_617)_(785_958)del, p.? (not in ClinVar, pathogenic)
18 (23) (2022)	14 y, male	NF1, delayed puberty, decreased visual acuity and headaches, gigantism (increased IGF-1, unsuppressed GH in OGTT). Underwent TSS. FH not mentioned	Pituitary MRI: 33x28x20 mm pituitary tumor with suprasellar extension, displacing the optic chiasm and extending along the cavernous sinus bilaterally	GH and PRL-expressing pituitary tumor	*NF1* NGS: *NF1:* c.1541A>C, p.Q514P (ClinVar VCV000232968.16, VUS), no LOH
19 (23) (2022)	42 y, female	NF1 (multiple skin neurofibromas, Lisch nodules), MNG, acromegaly (clinically and IGF-1 increased, no GH suppression in OGTT). Declined surgery and received medical treatment. FH not mentioned	Pituitary MRI: 14x10 mm pituitary tumor	Somatotropinoma?***	*NF1* NGS: *NF1:* c.2329T>A, p.W777R, (ClinVar VCV000230937.11, pathogenic/LP)
20 (71)**** (2023)	26 y, male	Acromegaly (clinically since age 18 y, IGF-1 1xULN but paradoxical GH increase in OGTT). Treatment not described. No NF1 manifestations, FH not mentioned	Pituitary MRI: lobulated sellar and suprasellar heterogeneously enhancing pituitary tumor. Ga-68 PET/CT: intense focus of uptake at the same location	Somatotropinoma?***	ES: *NF1*: c.1721+61_1721+63del, p.? (not in ClinVar, LP)
21 (this case)	54 y, male	See case description	See **Figures 1B–D**	See **Figure 2A**	NGS panel: heterozygous germline *NF1:* c.147C>A, p.Y49* (ClinVar: VCV002114679.2, pathogenic)
Somatic *NF1* variants					
22 (24) (2021)	59 y, male	Incidentally found PitNET (MRI done to plan a surgery to repair an aneurysm of the proximal ascending thoracic aorta). Elevated TSH. Treated with octreotide and then TSS. No NF1 manifestations, FH not mentioned	MRI: homogeneously enhancing bilobed sellar mass with suprasellar extension displacing the chiasm	Thyrotropinoma: diffuse PIT1 nuclear +, multiple TSH + cells, scattered SF1 + cells, and occasional PRL and GH + cells; Ki-67<3%	ES: heterozygous somatic *NF1*: c.305T>G, p.M102R (not in ClinVar, VUS). Also, increased CNV rate, reflected in alterations of chromosomes 2, 5, 7, 9, 14, 15, 18, 19, 20, and 21
23 (24) (2021)	46 y, male	Progressive loss of peripheral vision, normal neuroendocrine function. Underwent TSS with significant improvement of visual fields. Developed diabetes insipidus and central adrenal insufficiency. No NF1 manifestations, FH not mentioned	MRI: large, homogeneously enhancing giant sellar mass compressing the optic chiasm with extension into the third ventricle	Pituitary adenoma with diffuse nuclear positivity for SF1. Pituitary hormone expression not detected. Ki-67<3%	ES: heterozygous somatic *NF1*: c.3199G>T, p.D1067Y (ClinVar VCV000803351.1, LB)
24 (72) (2022)	53y, male	CD, treated with two TSS, RT, metyrapone, and ketoconazole, with persistent disease activity. No NF1 manifestations, FH not mentioned	Initial pituitary tumor size not reported. Postsurgical sellar MRI: no residual tumor. Thoracic and abdominal CT and craniospinal MRI: multiple lesions suspicious for metastases in the liver and in the body of thoracic vertebrae 2, 5, and 9	Metastatic ACTH-secreting PitNET: TPIT +, ATRX +. Liver metastases: 20 mitoses/10 HPF, ACTH +, TPIT -, Ki-67 60%, TP53 + in nearly all tumor cell nuclei, ATRX +	NGS panel: *NF1*: c.1318C>T, p.R440* (ClinVar VCV000230673.46, pathogenic) and heterozygous *TP53* (NM_000546.6): c.743G>A, p.R248Q (ClinVar VCV000012356.74, pathogenic) in PitNET and liver metastases. Heterozygous *PTEN* (NM_000314.8):c.388C>T, p.R130* and c.210-1G>A, p.? and *ATRX* (NM_000489.4): c.2044A>G, p.N682D in the liver tumor only. Sanger: no *USP8* and *USP48* hotspot variants in PitNET

*Age at diagnosis of PitNET. **Reference sequence for *NF1* variants: (NM_001042492.3); all *NF1* variants were found in heterozygosis.***Histopathological study not available. **** Only abstract available.

BEC, bromocriptine; CD, Cushing’s disease; ES, exome sequencing; FH, family history; FTC, follicular thyroid carcinoma; LB, likely benign; LP, likely pathogenic; MLPA, multiplex ligation-dependent probe amplification; MNG, multinodular goiter; NF2, neurofibromatosis type 2; ns, not specified; PGL, paraganglioma; PHEO, pheochromocytoma; PHTP, primary hyperparathyroidism; RT, radiotherapy; TSS, transsphenoidal surgery; VUS, variant of uncertain significance.

Not all tumors had a definitive clinical diagnosis due to historical difficulties in hormone assessment. Clinically, 13 (54%) of the patients exhibited acromegalic features, while 5 (21%) presented with compressive symptoms due to large tumor size. Four previously reported patients had tumors clearly categorized as clinically NF-PitNETs: two were a silent corticotropinomas, one was SF1 positive, and one had negative hormonal staining. One patient had a functioning thyrotropinoma, while another presented with hypercortisolism from a metastatic ACTH-secreting PitNET. Imaging was similarly challenging for the earliest cases due to limited techniques available at the time. In cases of PitNETs evaluated with MRI, the dimensions ranged from 5-7 mm for microadenomas and 10-33 mm for macroadenomas, with the latter often resulting in displacement of the optic chiasm. Only one patient with a germline *NF1* defect had also genetic testing of the tumor, but no LOH was found.

Germline *NF1* variants contribute to tumorigenesis by disrupting the negative regulation of the RAS/MAPK/ERK pathway, leading to enhanced mitotic activity and cellular proliferation (44). Since *NF1* is a tumor suppressor, the lack of LOH at the *NF1* defect locus in PitNETs from our patient and one previously studied individual (Case 18) may argue against a driver role for *NF1* in PitNETs. However, LOH is not a universal finding among NF1-associated tumors. A study of 91 classical non-endocrine NF1 tumors identified LOH in one-fifth of cases, with heterogeneous somatic hits among different tumors, particularly in cases where multiple lesions from the same individual were examined (45). In contrast, LOH is common in PPGLs, occurring both in cases with only somatic variants and in those with coexisting germline defects (10, 11, 46).

Alternative mechanisms could explain a possible causal association of *NF1* LOF and PitNETs. Firstly, additional defects could affect different regions of the *NF1* gene (47). This possibility cannot be ruled out in cases where only a short sequence was investigated in the tumor (Case 18 and ours). Secondly, as seen in other NF1 manifestations, *NF1* haploinsufficiency could be sufficient for the development of PitNETs (48). In line with this, recent studies in mouse models showed that *Nf1* ^+/-^ microenvironment accelerates the development of benign tumors while inhibiting their progression to malignancy (49). Furthermore, for haploinsufficient tumor suppressors, both underexpression (due to loss of one allele) and overexpression (as a compensatory response) can lead to imbalances in tightly regulated signaling pathways (50). An increased *NF1* expression could therefore create a paradoxical situation, resulting in toxicity, rather than physiological compensation.

Thirdly, somatic *NF1* variants could represent second hits in tumors with a different initial genetic insult. For instance, in a mouse strain with preexistent genomic instability and a high rate of breast cancer development, monoallelic or biallelic *Nf1* deletions occur in almost all tumors (51). Similarly, somatic *NF1* variants are frequent findings in multiple types of human cancers, often correlating with therapeutic resistance and increased aggressiveness (52). Unsurprisingly, at least one case of a PitNET harboring a somatic pathogenic *NF1* variant has been documented (Case 24). In this setting, it remains unclear whether the *NF1* sequence change represents a passenger or a driver defect.

Despite these data, the association of NF1 and PitNETs could be coincidental, given the frequent discovery of pituitary incidentalomas in the general population. Individuals with NF1 undergo brain imaging as part of their clinical surveillance, which could increase the risk for incidental pituitary lesions. Nevertheless, patients reported in **Table 2** exhibit a high incidence of GH-secreting and large PitNETs, whereas pituitary incidentalomas are most often small NF-PitNETs. Remarkably, 10 (45%) patients were <40 years at PitNET diagnosis. These characteristics (predominance of somatotropinomas and young onset) resemble those of PitNETs caused by proven causative germline defects (53).

A key limitation of this study is the absence of immunostaining for LH, FSH, and relevant transcription factors. The latter immunostainings are part of the current recommendations for PitNET classification (54), but are not available in most centers worldwide, including ours. Despite this, the most likely diagnosis remains a gonadotropinoma, consistent with the majority of NF-PitNET cases. While our review of NF1-associated PitNETs is informative, a larger cohort would provide a more robust understanding of the potential relationship between NF1 and PitNETs. The available data are insufficient to definitively confirm or refute causality, and it remains unclear whether *NF1* LOF plays an active role in pituitary tumorigenesis. Further investigations are required to elucidate the role of *NF1* variants in the pathogenesis of PitNETs.

## Conclusions

The association between NF1 and PitNETs represents a complex interplay that challenges our understanding of both conditions. While NF1 is traditionally associated with optic pathway gliomas and other neoplasms, the emergence of PitNETs in these patients adds a layer of clinical and genetic complexity. The case presented here highlights the need for further investigation into the genetic mechanisms underlying this association. Genetic testing revealed a pathogenic germline variant in *NF1*, suggesting a potential role in tumorigenesis. However, the absence of LOH in the PitNET points toward a nuanced genetic landscape that requires broader exploration, including deep clinical and genetic characterization of large cohorts of individuals with NF1. We were not able to determine a causal relationship between NF1 and the presence of a PitNET in this patient. Future studies will be essential for unraveling the mechanisms potentially linking NF1 and PitNETs, thereby guiding clinical management and genetic counseling strategies for affected individuals.

## Data Availability

The datasets presented in this study can be found in online repositories. The names of the repository/repositories and accession number(s) can be found below: https://www.ncbi.nlm.nih.gov/biosample/43250447, SAMN43250447.
